# Supplementary material for: Inflammasome-mediated GSDMD activation facilitates escape of Candida albicans from macrophages
Source: Nat Commun. 2021 Nov 18;12:6699. doi: 10.1038/s41467-021-27034-9 (PMC8602704; doi:10.1038/s41467-021-27034-9)
Supplement: Supplementary file 1 — Supplementary Information [file 41467_2021_27034_MOESM1_ESM.pdf]

Supplementary information

Inflammasome-mediated GSDMD activation facilitates escape of *Candida albicans* from macrophages

Xionghui Ding, Hiroto Kambara, Rongxia Guo, Apurva Kanneganti, Maikel Acosta-Zaldívar, Jiajia Li, Fei Liu, Ting Bei, Wanjun Qi, Xuemei Xie, Wenli Han, Ningning Liu, Cunling Zhang, Xiaoyu Zhang[1], Hongbo Yu, Li Zhao, Fengxia Ma, Julia R Köhler, and Hongbo R Luo

1. Supplementary Movies: Supplementary Movie 1-5

2. Supplementary Tables: Supplementary Table 1-4

3. Supplementary Figures: Supplementary Figure 1-10

4. Supplementary References

Supplementary Table 1. Key resources used in this study

REAGENT or RESOURCE	SOURCE	IDENTIFIER
**Antibodies**		
Anti-mouse GSDMD Antibody	Abcam	Cat# ab209845
Anti-mouse IL-1-β/IL-1F2 antibody	R&D Systems	Cat# AB-401-NA
Anti-mouse total IL-1β antibody	Cell Signaling	Cat# 12507
Anti-mouse cleaved-IL-1β (Asp117) antibody	Cell Signaling	Cat# 52718S
Anti-β-actin antibody	Sigma Aldrich	Cat# A2228
Candida albicans Polyclonal Antibody, FITC	Thermofisher	Cat# PA1-73154
Goat anti-Rabbit IgG (H+L) Secondary	Thermo Fisher	Cat# 65-6120
Goat anti-Mouse IgG (H+L) Secondary	Thermo Fisher	Cat# 62-6520
Mouse anti-goat IgG-HRP	Santa Cruz	Cat# sc-2354
Anti-mouse GAPDH (D16H11)	Cell Signaling	Cat# 5174S
Anti-mouse Caspase-1 (E2Z1C)	Cell Signaling	Cat# 24232S
Anti-mouse Cleaved Caspase-1 (Asp296)	Cell Signaling	Cat# 89332S
Anti-mouse Caspase-1 (p20)	AdipoGen	Cat# AG-20B-0042-C100
Anti-human Gasdermin D (E9S1X)	Cell Signaling	Cat# 39754S
Anti-human Cleaved Gasdermin D (Asp275)	Cell Signaling	Cat#36425S
Cleaved-IL-1β (Asp116) (D3A3Z)	Cell Signaling	Cat#83186S
Cleaved Caspase-1 (Asp297) (D57A2)	Cell Signaling	Cat#4199S
**Bacterial, fungal and Virus Strains**		
See **Supplementary Table 2** for *C. albicans* strains used in this study.	This study	N/A
**Chemicals, Peptides, and Recombinant Proteins**		
Propidium Iodide - 1.0 mg/mL Solution in	Thermo Fisher	Cat# P3566
2-mercaptoethanol	Bio-Rad	Cat# 1610710
2x Laemmli Sample Buffer	Bio-Rad	Cat# 1610737
10x Tris/Glycine/SDS	Bio-Rad	Cat# 1610772
10x Tris/Glycine Buffer for WBs and Native	Bio-Rad	Cat# 1610771
Blotting Grade Blocker Non-Fat Dry Milk	Bio-Rad	Cat# 1706404XTU
Fetal bovine serum	Gemini Bio-Products	Cat# S01520; Lot#A95E82G
Penicillin-Streptomycin (10,000 U/mL)	Thermo Fisher	Cat# 15140122
Recombinant Mouse M-CSF	Biolegend	Cat# 576406
Mouse Serum	Atlanta biologicals	Cat# S18193
SuperSignal™ Maximum Sensitivity	Thermo Fisher	Cat# 34096
Restore™ Western Blot Stripping Buffer	Thermo Scientific	Cat# 21059
DMEM	Thermo Fisher	Cat# 12430062
PBS	Thermo Fisher	Cat# 14190250
Opti-MEM® I Reduced Serum Medium	Thermo Fisher	Cat# 31985-062
PBS with Tween® 20 (PBST-20X)	Cell Signaling	Cat# 9809S
Protease Inhibitor Cocktail (100X)	Cell Signaling	Cat# 5871
Necrosulfonamide	R and D systems	Cat# 5025/50
Dimethyl sulfoxide	Sigma	Cat# D-2650
ACK Lysing buffer	Fisher/Gibco	Cat# A1049201
10% Tween 20	Bio-Rad	Cat# 1610781
Diisopropylfluorophosphate	Sigma	Cat# D0879
VX-765	Invivogen	Cat# inh-vx765i-5
Triton X-100	Sigma Aldrich	Cat# T8787

KCL	Thermo Fisher	Cat# AM9640G
IMDM, GlutaMAX Supplement	Thermo Fisher	Cat# 31980-030
EasySep™ Human Monocyte Isolation Kit	Stemcell technologies	Cat# #19359
Lymphoprep™	Stemcell technologies	Cat# 07801
Human M-CSF	Peprotech	Cat# AF-300-25
**Critical Commercial Assays**		
LDH Cytotoxicity Assay Kit	Thermo Fisher	Cat# 88954
Mouse IL-1 beta/IL-1F2 DuoSet ELISA	R&D Systems	Cat# DY401
Human IL-1 beta/IL-1F2 DuoSet ELISA	R&D Systems	Cat#DY201
**Deposited Data**		
N/A		
**Experimental Models: Cell Lines**		
N/A		
**Experimental Models: Organisms/Strains**		
Mouse: C57BL/6J		
**Recombinant DNA**		
See **Supplementary Table 3** for plasmids used in this study.	This study	N/A
**Oligonucleotides**		
See **Supplementary Table 4** for primer sequences	This study	N/A
**Other**		
Nitrocellulose Membrane	Bio-Rad	Cat# 1620146

Software and Algorithms

ImageJ	ImageJ	https://imagej.nih.gov/
ImageQuant LAS-	Cytiva	https://www.cytivalifesciences.com/en/us/support/products/imagequan
GraphPad Prism v8	GraphPa	https://www.graphpad.com/scientific-software/prism/
NIS-Elements	Nikon	https://www.nikon.com/products/microscope-
FlowJo software	BD	https://www.flowjo.com/solutions/flowjo/downloads/
cellSens Dimension	Olympu	https://www.olympus-lifescience.com/en/software/cellsens/

RESOURCE AVAILABILITY

Lead Contact

Further information and requests for resources and reagents should be directed to and will be fulfilled by the Lead Contact, Hongbo R. Luo (Hongbo.Luo@childrens.harvard.edu).

Materials Availability

Plasmids and *C. albicans* strains generated in this study are available from the Lead Contact with a completed Materials Transfer Agreement. This study did not generate any other unique distributable reagents.

Data and Code Availability

The published article includes all datasets generated or analyzed during this study. This study did not generate any other digitally accessible data or code.

Supplementary Table 2. ***C. albicans*** **strains used in this study**

Strain name	Parent	Genotype	Strain background /construction	Reference
SC5314		Wild type		Fonzi WA, Irwin MY (1993) Genetics [1]
CAF6-8		*Δura3::λimm434/Δura3::Δimm434 ecel:: hisG::I-SceII Δecel::hisG:JI-SceI-URA3-hisG::I-SceI*		Fonzi WA, Irwin MY (1993) Genetics [1]
HLC54		*ura3::1 imm434/ura3::1 imm434 cph1::hisG/cph1::hisG efg1::hisG/efg1::hisG-URA3-hisG*		Lo et al (1997) Cell [2]
JKC917	SN95	*hisΔ1/his1Δ:: tetR-FRT arg4/arg4 IRO1/iro1Δ::λimm^{434} URA3/ura3Δ::λimm^{434}*		Liu N-N et al (2017) Proc Natl Acad Sci U S A [3]
JKC1713	JKC917	*his1/his1:: tetR-FRT arg4/ARG4 IRO1/iro1Δ::λimm434 URA3/ura3Δ::λimm434*	JKC917 transformed with PCR product encoding *ARG4*, amplified with fjk1184 and rjk1186 using SC5314 (JKC302) genomic DNA as template. Integration of ARG4 gene verified by PCR with fjk1185 and rjk1187 (5' end), fjk1188 and rjk1199 (3' end).	This work
JKC2078	JKC1713	*pACT1-ACT1/pact1-act1::pACT1-GFP-pACT1-ACT1 his1/his1::tetR-FRT arg4/ARG4*	JKC1713 transformed with BsrGI digested pJK 1418. Integration of GFP encoding gene verified by PCR with fjk1514 and rjk1515 (5' end), fjk1517 and rjk1516 (3' end).	This work

Supplementary Table 3. Plasmids used in this study

Plasmid	Description	Source (Reference)
pJK1418	*GFP* encoding sequence was amplified with fjk1615 and rjk1633 using pJK184 (GFP-HIS1) as template. PCR product was ligated into pJK1085 using ClaI/XmaI sites.	This work
pJK184	pGFP-HIS1	Gerami-Nejad (2001) Yeast Functional Analysis Report [4]
pJK1085	Derived from pAU34 (ACT1 expression vector)	Uhl MA & Johnson AD (2001) Microbiology [5]

Supplementary Table 4. Primers used in this study

Primer name	Purpose	Sequence 5' to 3'
fjk1184	Amplification of *ARG4* gene, forward oligo	GAATCCACAATCGTATATGAAC
rjk1186	Amplification of *ARG4* gene, reverse oligo	GAATATAGTGATGATGAGGATG
fjk1185	Verification of *ARG4* gene integration on the 5' end, forward oligo	GACATATTGACCGACATAATTC
rjk1187	Verification of *ARG4* gene integration on the 5' end, reverse oligo	GTCGTTTCACCGGTGCCACTG
fjk1188	Verification of *ARG4* gene integration on the 3' end, forward oligo	CAGTACCACCAATAGCATCTC
rjk1199	Verification of *ARG4* gene integration on the 3' end, reverse oligo	GGTAGTCTCCGATTATGATTC
fjk1615	Amplification of *GFP* encoding gene, forward oligo	CCTGCTatcgatATGTCTAAAGGTGAAGAATTAT
rjk1633	Amplification of *GFP* encoding gene, reverse oligo	GCAGCTcccgggTTATTTGTATAATTCATCCATACCATGG
fjk1514	5' end verification of *GFP* gene integration in the *ACT1* locus, forward oligo	GACACCTAACTAATAAACC
rjk1515	5' end verification of *GFP* gene integration in the *ACT1* locus, reverse oligo	CCAGTAAATAATTCTTCACC
fjk1517	3' end verification of *GFP* gene integration in the *ACT1* locus, forward oligo	GGAATTGTGAGCGGATAAC
rjk1516	3' end verification of *GFP* gene integration in the *ACT1* locus, reverse oligo	CAATCAATTAGAATTGAAGC

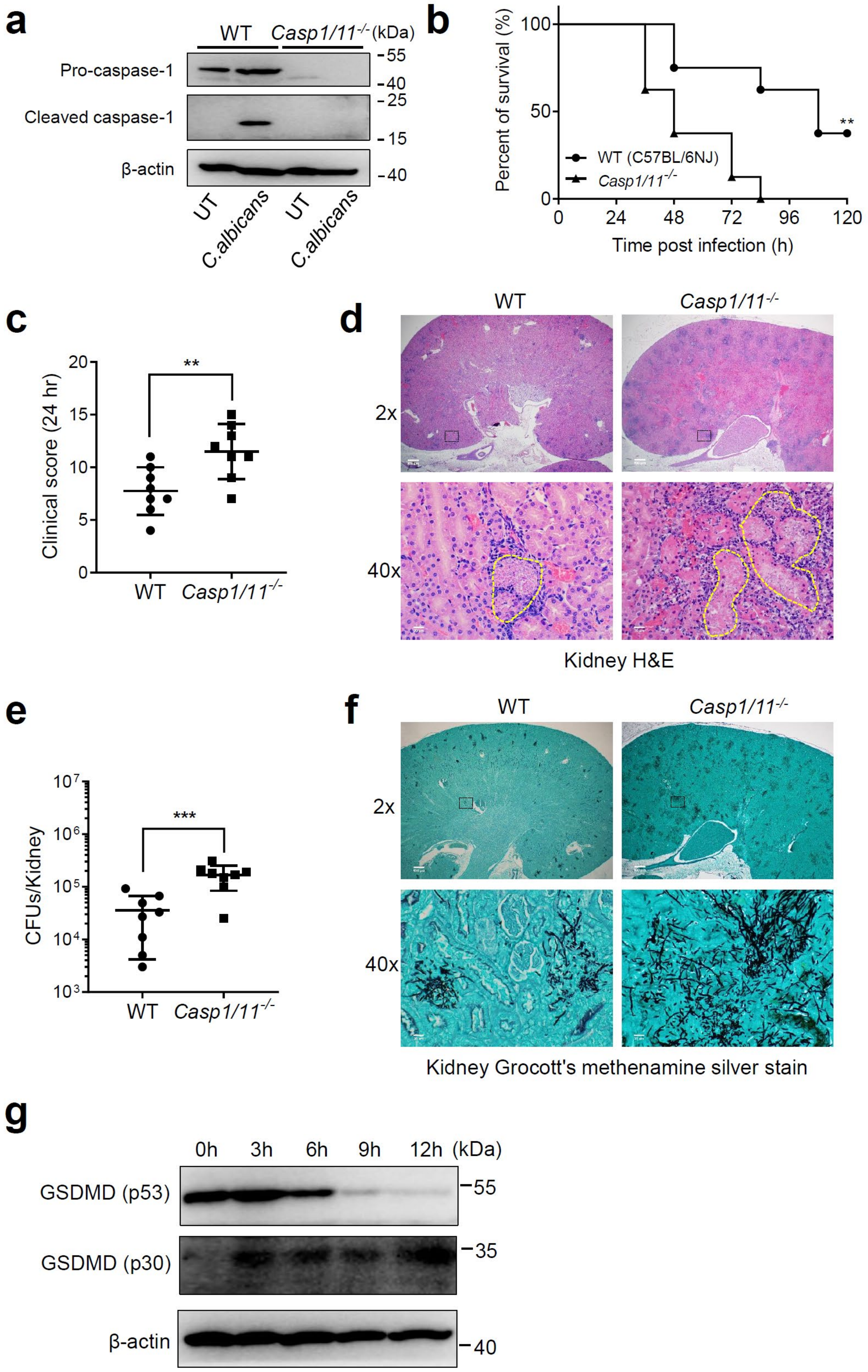

a
WT
Casp1/11-/-
(kDa)
Pro-caspase-1
Cleaved caspase-1
β-actin
55
40
25
15
40
UT
C.albicans
UT
C.albicans
b
Percent of survival (%)
100
50
0
0
24
48
72
96
120
Time post infection (h)
WT (C57BL/6NJ)
Casp1/11-/-
**
c
Clinical score (24 hr)
20
15
10
5
0
WT
Casp1/11-/-
**
d
WT
Casp1/11-/-
2x
40x
Kidney H&E
e
CFUs/Kidney
10^7
10^6
10^5
10^4
10^3
WT
Casp1/11-/-
***
f
WT
Casp1/11-/-
2x
40x
Kidney Grocott's methenamine silver stain
g
0h
3h
6h
9h
12h
(kDa)
GSDMD (p53)
GSDMD (p30)
β-actin
55
35
40

Supplementary Figure 1.

(a-f) *Casp1/11* disruption aggravates *C. albicans* infection.

(a) Caspase-1 expression was abolished in *Casp-1/11*$^{-/-}$ mice. BMDMs from WT (C57BL/6J) or in *Casp-1/11*$^{-/-}$ mice were untreated or infected with WT *C. albicans* for 6 h at MOI 50. Pro-caspase-1 and cleaved caspase-1 in the cell lysates were detected by western blotting. The figure shows the result of a representative experiment that was repeated three times. Source data are provided as a Source Data file.

(b) Kaplan-Meier survival plots of *C. albicans*-challenged WT (C57BL/6NJ) and *Casp1/11*$^{-/-}$ (C57BL/6NJ) mice. Age- and sex-matched (10-week-old female) WT and *Casp1/11*$^{-/-}$ mice were intravenously challenged with 1×10^6 CFU *C. albicans*. Survival rates were analyzed using Kaplan-Meier survival curves and log-rank testing. Two-tailed unpaired Student's t test was used for statistical comparisons. Data shown are means ±SD (n=8 mice per group). p=0.0041 *vs.* WT.

(c) Clinical scores of *C. albicans*-challenged WT and *Casp1/11*$^{-/-}$ mice. Two-tailed unpaired Student's t test was used for statistical comparisons. Data shown are means ±SD (n=8 mice per group). p=0.0083 *vs.* WT.

(d) Histopathologic assessment of the kidneys of WT and *Casp1/11*$^{-/-}$ mice 2 days after intravenous injection of 1×10^6 CFU *C. albicans*. Shown are representative H&E-stained sections of kidney tissues. Experiments were repeated three times.

(e) Fungal burden of kidneys 2 days after *C. albicans* infection. Two-tailed unpaired Student's t test was used for statistical comparisons. Data shown are means ±SD (n=8 mice per group). p=0.0009 *vs.* WT.

(f) *C. albicans* in the kidney were identified by Grocott methenamine silver staining. Mice were sacrificed 2 days after intravenous injection of 1×10^6 CFU *C. albicans*. Results are representative of data from at least three biological replicates.

(g) *C. albicans*-triggered GSDMD cleavage in mouse bone marrow-derived macrophages (BMDMs). BMDMs were infected with *C. albicans* for indicated time periods at MOI 10. Full-length (p53) GSDMD and cleaved (p30) GSDMD in the cell lysates were detected by western blotting as described in **Fig.1a**. Source data are provided as a Source Data file.

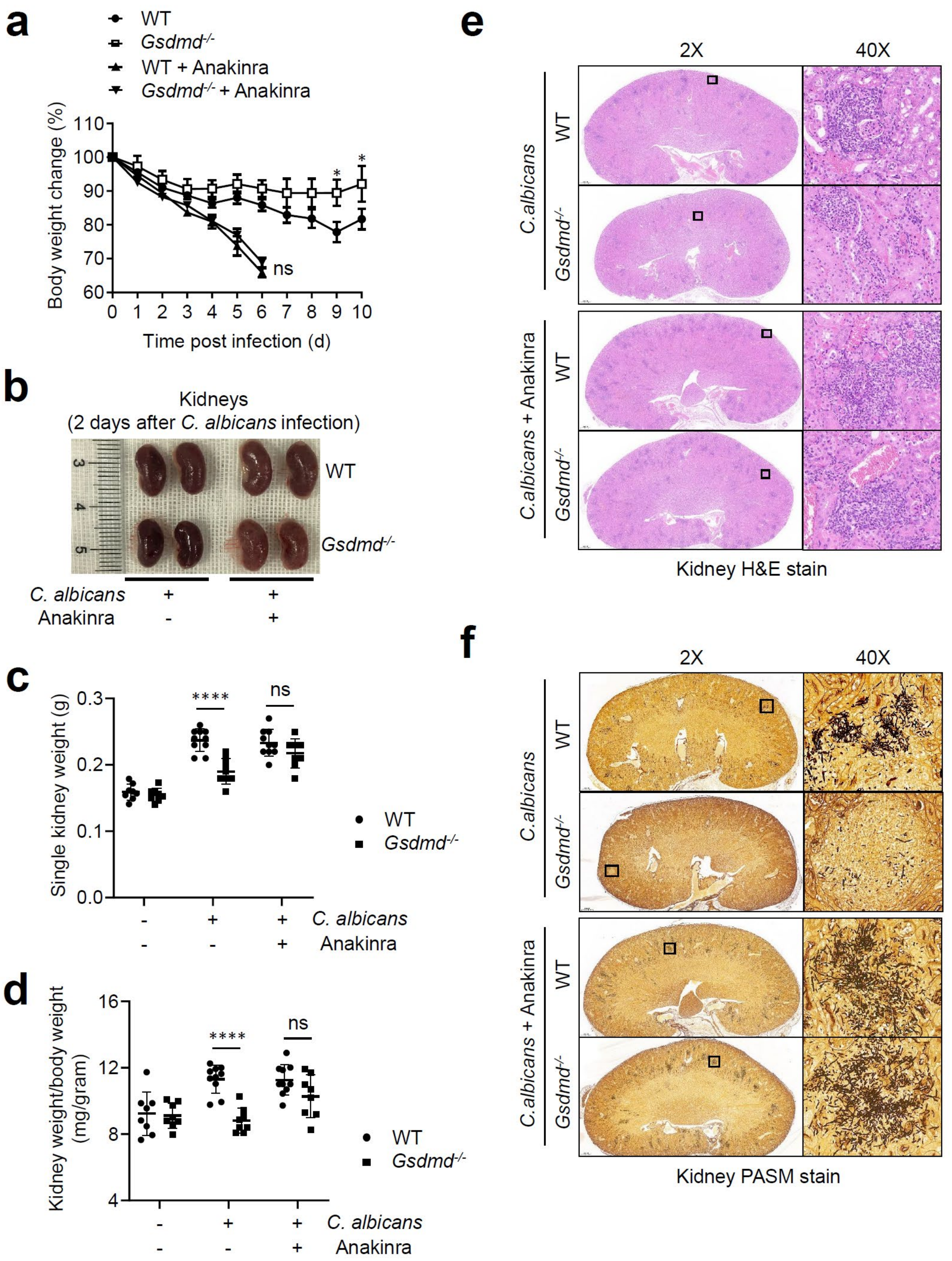
a
WT
Gsdmd-/-
WT + Anakinra
Gsdmd-/- + Anakinra
Body weight change (%)
110
100
90
80
70
60
*
*
ns
0 1 2 3 4 5 6 7 8 9 10
Time post infection (d)
b
Kidneys
(2 days after C. albicans infection)
WT
Gsdmd-/-
C. albicans + +
Anakinra - +
c
Single kidney weight (g)
0.3
0.2
0.1
0.0
****
ns
WT
Gsdmd-/-
- + + C. albicans
- - + Anakinra
d
Kidney weight/body weight (mg/gram)
16
12
8
4
****
ns
WT
Gsdmd-/-
- + + C. albicans
- - + Anakinra
e
2X
40X
C.albicans
WT
Gsdmd-/-
C.albicans + Anakinra
WT
Gsdmd-/-
Kidney H&E stain
f
2X
40X
C.albicans
WT
Gsdmd-/-
C.albicans + Anakinra
WT
Gsdmd-/-
Kidney PASM stain

Supplementary Figure 2. The protective effect against *C. albicans* infection triggered by GSDMD disruption is abolished by treatment with anakinra, an interleukin 1 receptor antagonist.

(a) Comparison of body weight changes of untreated or anakinra-treated WT and *Gsdmd*$^{-/-}$ mice. Age- and sex-matched WT and *Gsdmd*$^{-/-}$ mice were intravenously challenged with 2×10^5 CFU *C. albicans.* Anakinra was administrated 1h before *C. albicans* injection and then 23h after *the* injection. Two-tailed unpaired Student's t test was used for statistical comparisons. Data presented are the means ± SD of 3 independent experiments. n=9 for WT (untreated), n=4 for *Gsdmd*$^{-/-}$ (untreated), n=10 for WT (anakinra-treated), n=10 for *Gsdmd*$^{-/-}$ (anakinra-treated). For untreated mice, p=0.0338 (day 9), p=0.0443 (day 10) *vs.* WT. For anakinra-treated mice, p=0.2475 (day 6) *vs.* WT.

(b) Comparison of the kidneys of *C. albicans*-challenged untreated or anakinra-treated WT and *Gsdmd*$^{-/-}$ mice. Mice were sacrificed 2 days after intravenous injection of 1×10^6 CFU *C. albicans*. Shown are representative images from three independent experiments.

(c) Kidney weights of *C. albicans*-challenged untreated or anakinra-treated WT and *Gsdmd*$^{-/-}$ mice. Two-tailed unpaired Student's t test was used for statistical comparisons. Data presented are the means ± SD (n=8 mice per data point) of three independent experiments. ****p<0.0001 (without anakinra), p=0.1367 (with anakinra) *vs.* WT.

(d) Kidney weights are shown as the weight per gram body weight of each mouse. Two-tailed unpaired Student's t test was used for statistical comparisons. Data presented are the means ± SD (n=8 mice per data point) of three independent experiments. ****p<0.0001 (without anakinra), p=0.0771 (with anakinra) *vs.* WT.

(e) Histopathologic assessment of the kidneys of *C. albicans*-challenged untreated or anakinra-treated WT and *Gsdmd*$^{-/-}$mice. Mice were sacrificed 2 days after intravenous injection of 1 x 10^6 CFU *C. albicans*. Shown are representative H&E-stained sections of kidney tissues. Experiments were repeated three times.

(f) *C. albicans* in the kidney were identified by periodic acid-silver methenamine (PASM) staining. Mice were sacrificed 2 days after intravenous injection of 1×10^6 CFU *C. albicans*. Results are representative of data from at least three biological replicates.

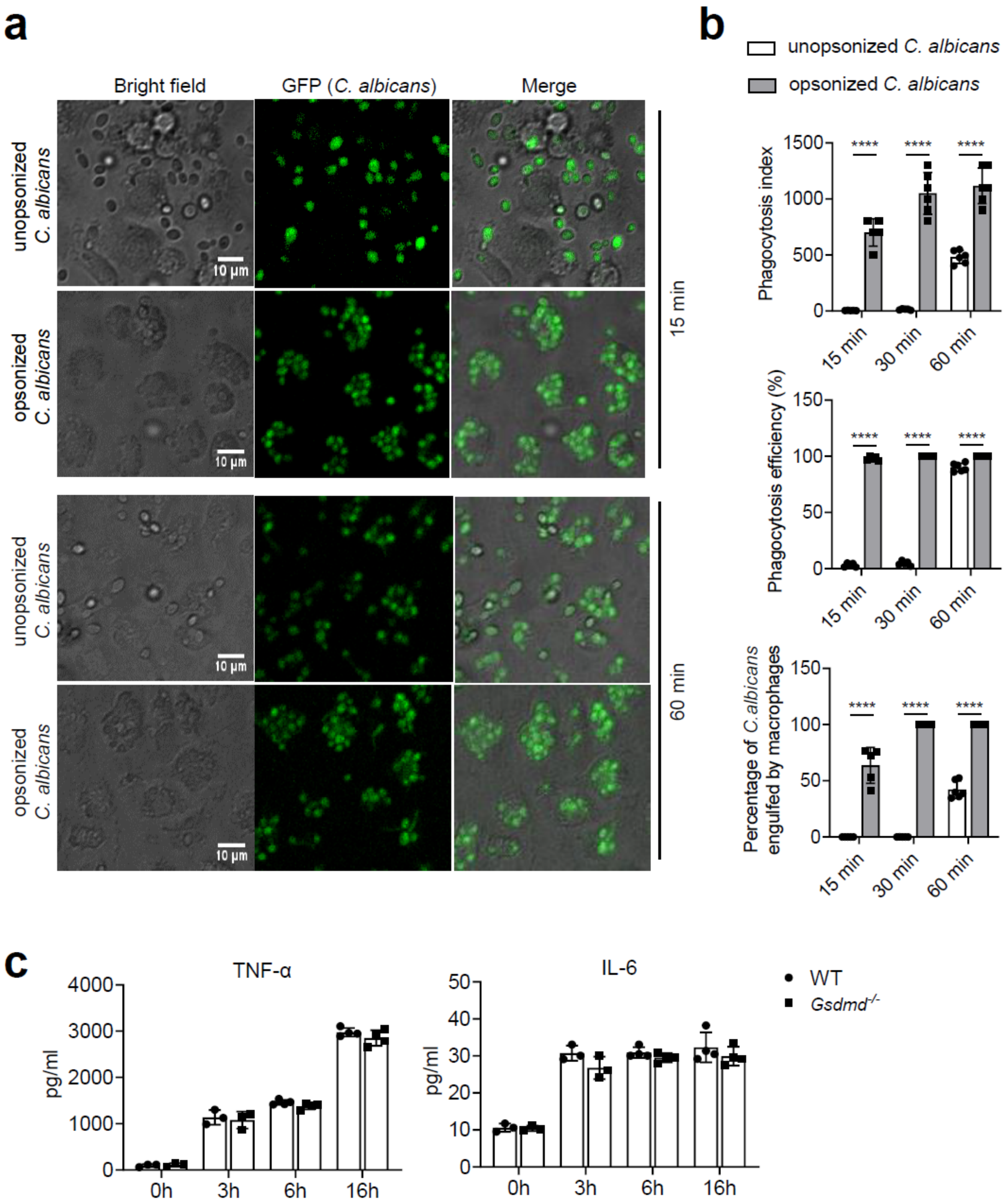
a
Bright field
GFP (C. albicans)
Merge
unopsonized C. albicans
opsonized C. albicans
10 μm
15 min
60 min
b
unopsonized C. albicans
opsonized C. albicans
Phagocytosis index
Phagocytosis efficiency (%)
Percentage of C.albicans engulfed by macrophages
****
15 min
30 min
60 min
c
TNF-α
IL-6
pg/ml
0h
3h
6h
16h
WT
Gsdmd-/-

Supplementary Figure 3.

(a-b) Serum-opsonised *C. albicans* are phagocytosed more efficiently than unopsonized *C. albicans.*

(a) WT BMDMs were incubated with GFP-expressing *C. albicans* at MOI 10 for indicated time. Shown are the representative images. Scale bars represent 10 μm.

(b) Quantification of *in vitro* phagocytosis capacity of BMDMs. Phagocytosis efficiency was expressed as the percentage of BMDMs that engulfed at least one *C. albicans*. Phagocytosis index was expressed as the average number of internalized *C. albicans* per 100 cells. At least 200 cells were assessed for each sample. Two-tailed unpaired Student's t test was used for statistical comparisons. Data shown are means ±SD (n=5 for 15 and 30min, n=6 for 60min). $p<0.0001$ *vs.* unopsonized *C. albicans*.

(c) *Gsdmd* disruption does not affect *C. albicans*-induced production of TNFα or IL-6.

BMDMs were infected with *C. albicans* (MOI=10) for the indicated time periods. TNFα and IL-6 in culture supernatants was measured by ELISA. Data shown are means ±SD (n=3 biologically independent samples per group). Experiments were repeated three times.

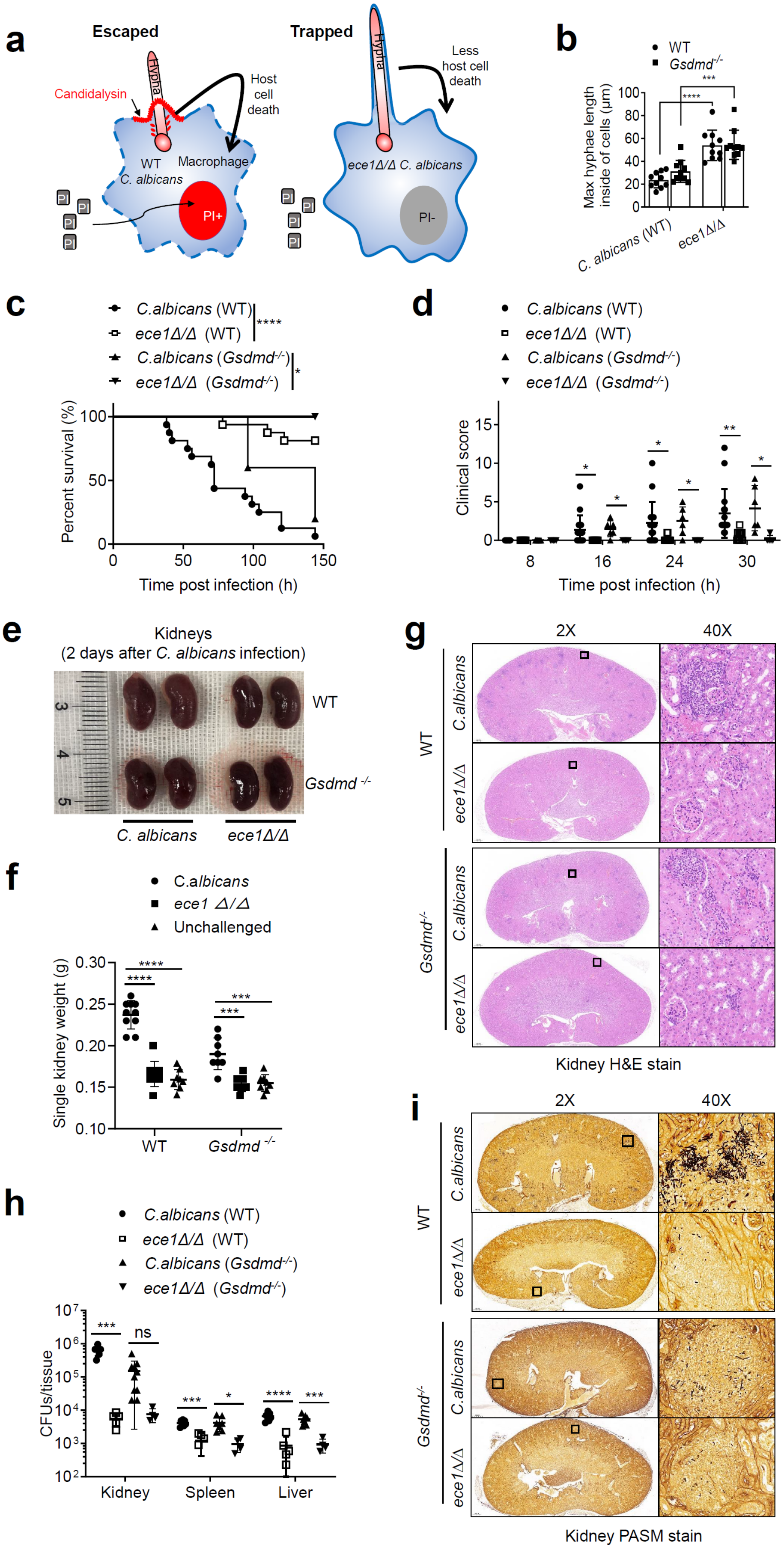
a
Escaped
Trapped
Candidalysin
Hypha
Host cell death
WT C. albicans
Macrophage
PI
PI+
Hypha
Less host cell death
ece1Δ/Δ C. albicans
PI-
b
WT
Gsdmd-/-
Max hyphae length inside of cells (μm)
C. albicans (WT)
ece1Δ/Δ
c
C.albicans (WT)
ece1Δ/Δ (WT)
C.albicans (Gsdmd-/-)
ece1Δ/Δ (Gsdmd-/-)
Percent survival (%)
Time post infection (h)
d
C.albicans (WT)
ece1Δ/Δ (WT)
C.albicans (Gsdmd-/-)
ece1Δ/Δ (Gsdmd-/-)
Clinical score
Time post infection (h)
e
Kidneys
(2 days after C. albicans infection)
WT
Gsdmd -/-
C. albicans
ece1Δ/Δ
f
C.albicans
ece1 Δ/Δ
Unchallenged
Single kidney weight (g)
WT
Gsdmd -/-
g
2X
40X
WT
Gsdmd-/-
C.albicans
ece1Δ/Δ
Kidney H&E stain
h
C.albicans (WT)
ece1Δ/Δ (WT)
C.albicans (Gsdmd-/-)
ece1Δ/Δ (Gsdmd-/-)
CFUs/tissue
ns
Kidney
Spleen
Liver
i
2X
40X
WT
Gsdmd-/-
C.albicans
ece1Δ/Δ
Kidney PASM stain

Supplementary Figure 4.

(a-b) Candidalysin facilitates *C. albicans*-induced rupture of the plasma membrane and macrophage lytic death, which enable *C. albicans* to escape from host cells.

(a) A schematic diagram illustrating the role of candidalysin in mediating macrophage death and facilitating *C. albicans* escape from macrophages.

(b) The length of candida hyphae in macrophages. The experiment was conducted as described in **Fig.6a**. Shown are the maximum lengths of intracellular hyphae in WT and *Gsdmd*$^{-/-}$ macrophages. At least 100 cells were assessed for each sample. Two-tailed unpaired Student's t test was used for statistical comparisons. Data shown are means ±SD (n=10 biological independent samples per group). $p<0.0001$ (*ece1Δ/Δ*-treated WT macrophages), $p=0.0002$ (*ece1Δ/Δ*-treated *Gsdmd*$^{-/-}$ macrophages) *vs.* WT *C. albicans*.

(c-i) Candidalysin-deficient *C. albicans* causes less severe infection and tissue damage in *Gsdmd*$^{-/-}$ mice.

(c) Kaplan-Meier survival plots of mice infected with WT or candidalysin-deficient (*ece1Δ/Δ*) *C. albicans*. Mice were intravenously challenged with 1×10^6 CFU *C. albicans*. Survival rates were analyzed using Kaplan-Meier survival curves and log-rank testing. Data shown are means ±SD (n=16 WT mice per group, n=5 *Gsdmd*$^{-/-}$ mice per group). $p<0.0001$ (WT mice), $p=0.0144$ (*Gsdmd*$^{-/-}$ mice) *vs.* WT *C. albicans*.

(d) Clinical scores of mice challenged with WT or candidalysin-deficient (*ece1Δ/Δ*) *C. albicans*. Two-tailed unpaired Student's t test was used for statistical comparisons. Data shown are means ±SD (n=16 WT mice per group, n=5 *Gsdmd*$^{-/-}$ mice per group). $p=0.029$ (16h WT mice), $p=0.0144$ (16h *Gsdmd*$^{-/-}$ mice), $p=0.0287$ (24h WT mice), $p=0.0159$ (24h *Gsdmd*$^{-/-}$ mice), $p=0.087$ (30h WT mice), $p=0.0156$ (30h *Gsdmd*$^{-/-}$ mice) *vs.* WT *C. albicans*.

(e) Comparison of the kidneys of mice challenged with WT or candidalysin-deficient (*ece1Δ/Δ*) *C. albicans*. Mice were sacrificed 2 days after intravenous injection of 1×10^6 CFU *C. albicans*. Shown are representative images.

(f) Kidney weights of *C. albicans*-challenged mice. Two-tailed unpaired Student's t test was used for statistical comparisons. Data shown are means ±SD (n=16-18 kidneys for WT group, n=8 kidneys for *Gsdmd*$^{-/-}$ group). For WT mice, $p<0.0001$ (*ece1Δ/Δ,* unchallenged) *vs.* WT *C. albicans*. For *Gsdmd*$^{-/-}$ mice, $p=0.0003$ (*ece1Δ/Δ*), $p=0.0004$ (unchallenged) *vs.* WT *C. albicans*.

(g) Histopathologic assessment of the kidneys of mice challenged with WT or candidalysin-deficient (*ece1Δ/Δ*) *C. albicans*. Mice were sacrificed 2 days after intravenous injection of 1×10^6 CFU *C. albicans*. Shown are representative H&E-stained sections of kidney tissues. Experiments were repeated three times.

(h) Fungal burden of kidneys, livers, and spleens of mice challenged with WT or candidalysin-deficient (*ece1Δ/Δ*) *C. albicans*. Mice were sacrificed 2 days after intravenous injection of 1×10^6 CFU *C. albicans*. Two-tailed unpaired Student's t test was used for statistical comparisons. Data shown are means ±SD (n=12 for *C. albicans*-treated WT mice, n=13 for *ece1Δ/Δ*-treated WT mice, n=11 for *C. albicans*-treated *Gsdmd*$^{-/-}$ mice, n=4 for *ece1Δ/Δ*-treated *Gsdmd*$^{-/-}$mice). $p<0.0001$ (WT kidney), $p=0.0837$ (*Gsdmd*$^{-/-}$ kidney), $p<0.0001$ (spleen), $p<0.0001$ (WT liver), $p=0.0008$ (30h *Gsdmd*$^{-/-}$ liver) *vs.* WT *C. albicans*.

(i) *C. albicans* in the kidney were identified by periodic acid-silver methenamine (PASM) staining. Mice were sacrificed 2 days after intravenous injection of 1×10^6 CFU *C. albicans*. Results are representative of data from at least three biological replicates.

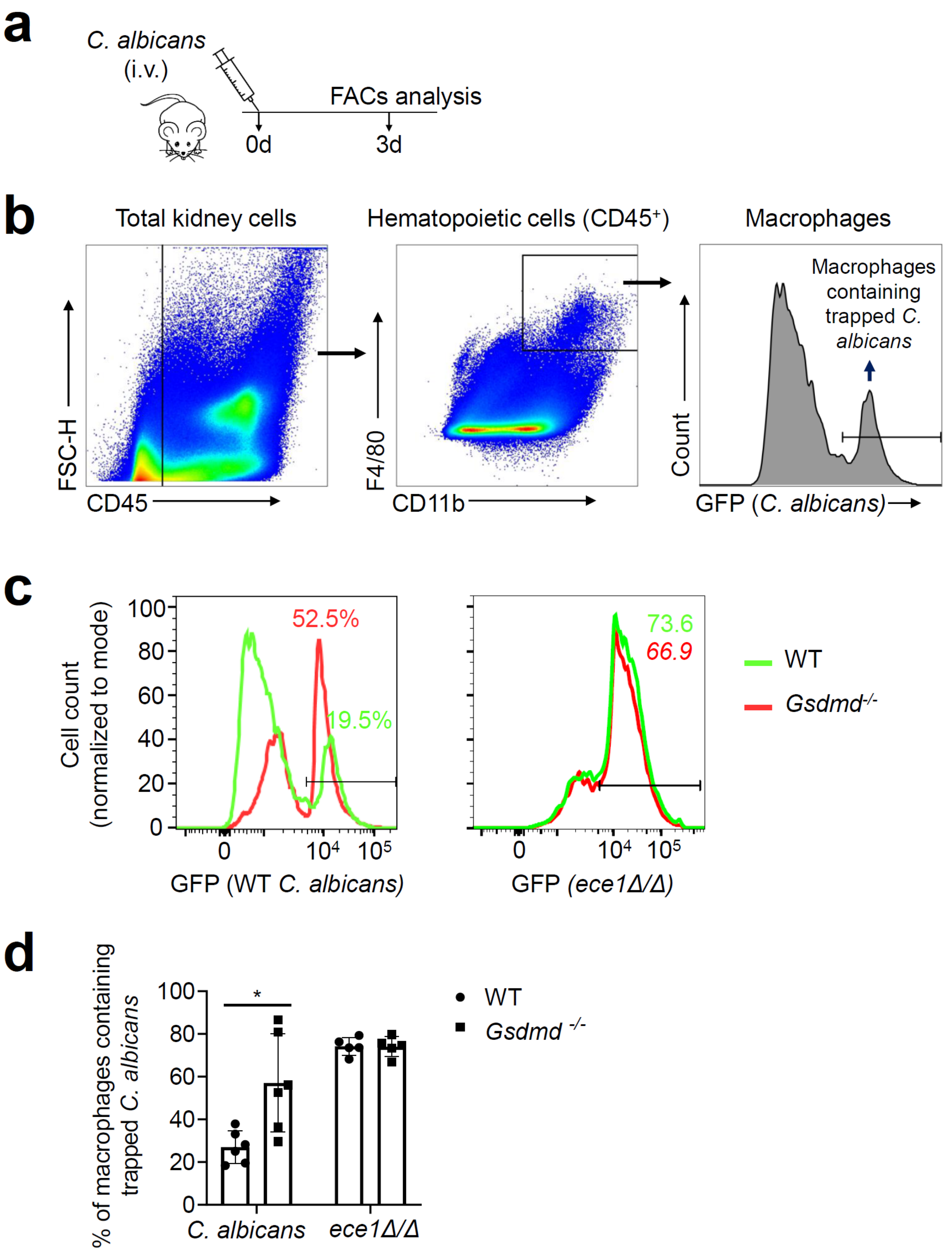

a
C. albicans (i.v.)
FACs analysis
0d
3d
b
Total kidney cells
Hematopoietic cells (CD45+)
Macrophages
FSC-H
CD45
F4/80
CD11b
Count
GFP (C. albicans)
Macrophages containing trapped C. albicans
c
Cell count (normalized to mode)
52.5%
19.5%
GFP (WT C. albicans)
73.6
66.9
GFP (ece1Δ/Δ)
WT
Gsdmd-/-
d
% of macrophages containing trapped C. albicans
*
C. albicans
ece1Δ/Δ
WT
Gsdmd -/-

Supplementary Figure 5. Flow-cytometry assessment of macrophages containing trapped *C. albicans* in the kidney.

(a) Experimental scheme. Mice (WT and *Gsdmd*$^{-/-}$) were intravenously challenged with 3×10^5 CFU WT (GFP$^+$) or candidalysin-deficient (ece1Δ/Δ) *C. albicans*. Kidney cells were harvested for flow cytometry analysis 3 days after the infection.

(b) Gating strategy of flow cytometry.

(c) Representative flow cytometric analysis of macrophages containing trapped *C. albicans* in WT (green lines) or *Gsdmd*$^{-/-}$ (red lines) mice. Macrophages containing trapped *C. albicans* were recognized by GFP fluorescence.

(d) The percentage of macrophages containing trapped *C. albicans* in WT or *Gsdmd*$^{-/-}$ mice infected with WT or candidalysin-deficient (*ece1Δ/Δ*) *C. albicans*. Two-tailed unpaired Student's t test was used for statistical comparisons. Data shown are means ±SD (n=6 for *C. albicans* treated mice, n=5 for *ece1Δ/Δ* treated mice). p=0.0126 (*C. albicans*) *vs.* WT.

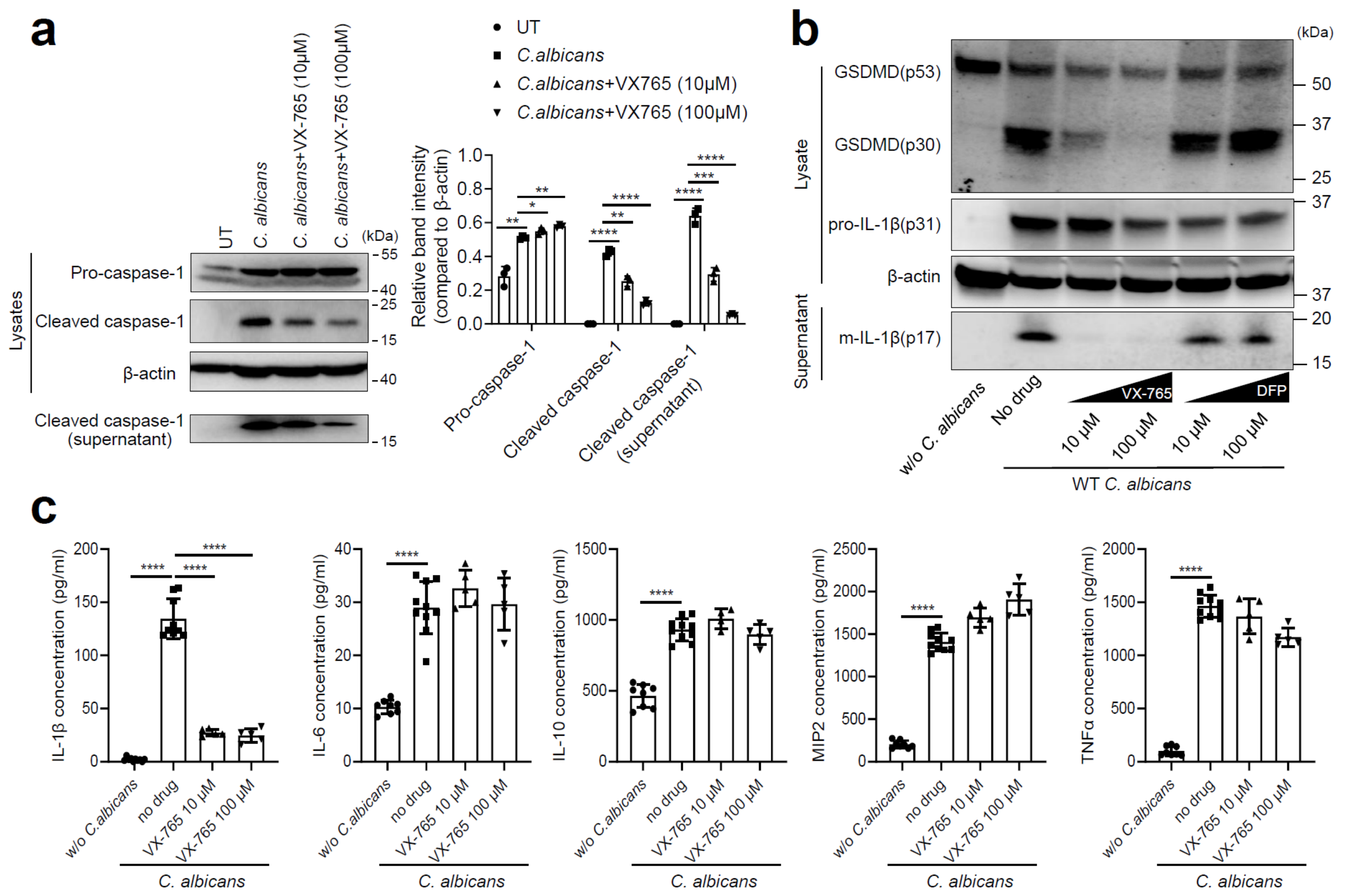

Supplementary Figure 6.

(a) GSDMD cleavage was blocked by VX-765. BMDMs were treated with caspase-1 inhibitor VX-765 for 1 h and then infected with *C. albicans* for 6 h at MOI 50. Pro-caspase-1 and cleaved caspase-1 in the cell lysates, and cleaved caspase-1 in supernatants were detected by western blotting. The figure shows the result of a representative experiment that was repeated three times. The intensities of protein bands were quantified using ImageJ. Two-tailed unpaired Student's t test was used for statistical comparisons. Data shown are means ±SD (n=3 biologically independent samples per group). For pro-caspase-1, p=0.0024 (UT), p=0.0363 (VX765 10μM), p=0.001 (VX765 100μM) *vs. C. albicans* alone treated. For cleaved-caspase-1 in lysate, p<0.0001 (UT), p=0.0013 (VX765 10μM), p<0.0001 (VX765 100μM) *vs. C. albicans* alone treated. For cleaved-caspase-1 in supernatant, p<0.0001 (UT), p=0.0006 (VX765 10μM), p<0.0001 (VX765 100μM) *vs. C. albicans* alone treated. Source data are provided as a Source Data file.

(b) *C. albicans*-induced GSDMD cleavage is mediated by caspases but not serine proteases. BMDMs were treated with caspase-1 inhibitor VX-765 or serine protease inhibitor diisopropylfluorophosphate (DFP) for 1 h and then infected with *C. albicans* for 6 h at MOI 50. GSDMD (cleaved p30 and full-length p53), pro-IL-1β in the cell lysates, and m-IL-1β in supernatants were assessed by western blotting. Results are representative of data from at least three biological replicates. Source data are provided as a Source Data file.

(c) Treatment with VX-765 specifically suppressed *C. albicans*-induced IL-1β production. BMDMs were infected with *C. albicans* (MOI=10) for 12 h in the presence or absence of VX-765. IL-1β, IL-10, MIP2, TNFα and IL-6 in culture supernatants were measured by ELISA. Two-tailed unpaired Student's t test was used for statistical comparisons. Data shown are means ±SD (n=8 for w/o *C. albicans*, n=10 for no drug, n= for VX765 treated samples). p<0.0001 *vs.* no drug treated.

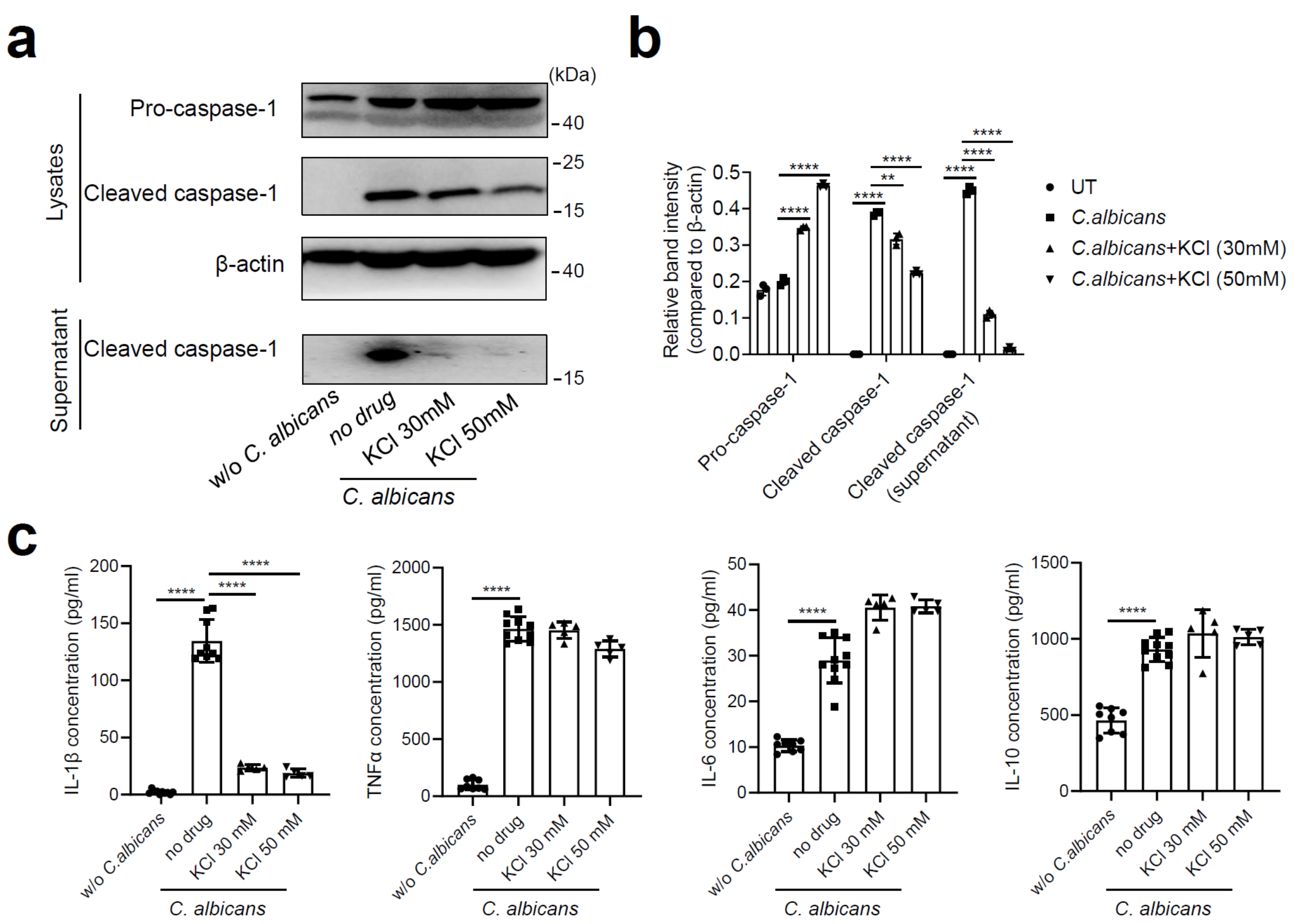

Supplementary Figure 7.

(a-b) Potassium efflux was essential for efficient *C. albicans*-triggered pro-caspase-1 cleavage in BMDMs.

(a) BMDMs were cultured in the indicated concentrations of KCl for 1 h and then infected with WT *C. albicans* for 6 h at MOI 50. Pro-caspase-1 and cleaved caspase-1 in the cell lysates, and cleaved caspase-1 in supernatants were detected by western blotting. The figure shows the result of a representative experiment that was repeated three times. Source data are provided as a Source Data file.

(b) The intensities of protein bands were quantified using ImageJ. Two-tailed unpaired Student's t test was used for statistical comparisons. Data shown are means ±SD (n=3 biologically independent samples per group). For pro-caspase-1, p<0.0001 (KCl 30/50mM) *vs. C. albicans* alone treated. For cleaved-caspase-1 in lysate, p<0.0001 (UT, KCl 50mM), p=0.0018 (KCl 30mM) *vs. C. albicans* alone treated. For cleaved-caspase-1 in supernatant, p<0.0001 (UT, KCl 30mM, KCl 50mM) *vs. C. albicans* alone treated.

(c) Treatment with KCl specifically suppressed *C. albicans*-induced IL-1β production. BMDMs were infected with *C. albicans* (MOI=10) for 12 h in the presence or absence of KCl. IL-1β, IL-10, TNFα and IL-6 in culture supernatants were measured by ELISA. Two-tailed unpaired Student's t test was used for statistical comparisons. Data shown are means ±SD (n=8 for w/o *C. albicans*, n=10 for no drug, n=5 for KCl-treated samples). p<0.0001 *vs.* no drug treated.

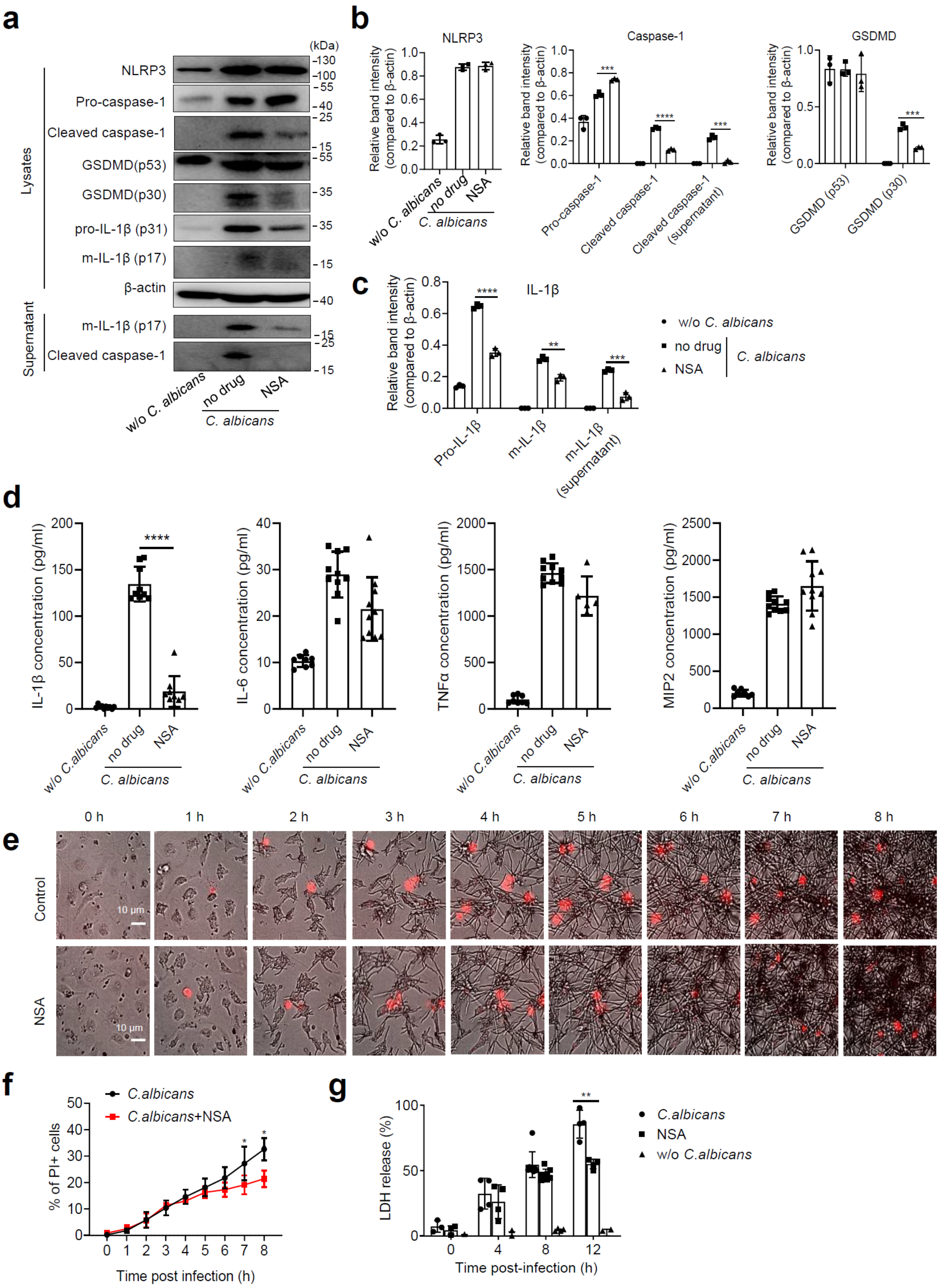

a
b
c
d
e
f
g
NLRP3
Pro-caspase-1
Cleaved caspase-1
GSDMD(p53)
GSDMD(p30)
pro-IL-1β (p31)
m-IL-1β (p17)
β-actin
Lysates
Supernatant
w/o C. albicans
no drug
NSA
C. albicans
(kDa)
Caspase-1
GSDMD
IL-1β
Relative band intensity (compared to β-actin)
IL-1β concentration (pg/ml)
IL-6 concentration (pg/ml)
TNFα concentration (pg/ml)
MIP2 concentration (pg/ml)
0 h
1 h
2 h
3 h
4 h
5 h
6 h
7 h
8 h
Control
10 μm
C.albicans
C.albicans+NSA
% of PI+ cells
Time post infection (h)
LDH release (%)
Time post-infection (h)

Supplementary Figure 8. GSDMD antagonist NSA alleviates *C. albicans*-induced death of mouse BMDMs.

(a) NSA suppressed *C. albicans*-triggered GSDMD cleavage in BMDMs. NSA treated and untreated BMDMs were infected with WT *C. albicans* for 6 h at MOI 50. Full-length (p53) GSDMD, cleaved (p30) GSDMD, NLRP3, pro-IL-1β, mature IL-1β (m-IL-1β), pro-caspase-1, cleaved caspase-1and actin in the cell lysates, as well as m-IL-1β and cleaved caspase-1 in the supernatant, were detected by western blotting. The figure shows the result of a representative experiment that was repeated three times. Source data are provided as a Source Data file.

(b) The intensities of indicated protein bands were quantified using ImageJ. Two-tailed unpaired Student's t test was used for statistical comparisons. Data shown are means ±SD (n=3 biologically independent samples). p=0.0006 (pro-caspase-1), p<0.0001 (cleaved-caspase-1 in lysate), p=0.0001 (cleaved-caspase-1 in supernatant), p=0.0003(GSDMD (p30)) *vs. C. albicans* alone treated.

(c) The intensities of pro-IL-1β and m-IL-1β protein bands were quantified using ImageJ. Two-tailed unpaired Student's t test was used for statistical comparisons. Data shown are means ±SD (n=3 biologically independent samples). p<0.0001 (pro-IL-1β), p=0.0013 (m-IL-1β in lysate), p=0.0004 (m-IL-1β in supernatant) *vs.* untreated (no drug).

(d) Treatment with NSA specifically suppressed *C. albicans*-induced IL-1β production. BMDMs were infected with *C. albicans* (MOI=10) for 12 h in the presence or absence of NSA. IL-1β, MIP2, TNFα and IL-6 in culture supernatants were measured by ELISA. Two-tailed unpaired Student's t test was used for statistical comparisons. Data shown are means ±SD (n=10 biologically independent samples per group). p<0.0001 (IL-1β) *vs. C. albicans* alone treated.

(e) NSA treated and untreated BMDMs were incubated with *C. albicans* at MOI 10. Dying cells were stained with propidium iodide (PI) (red) (200 ng/mL). Images were acquired at indicated time points using 20× dry lens. Shown are representative images at the indicated time points. Scale bars represent 10 µm. Experiments were repeated three times.

(f) The percentage of PI-positive BMDMs calculated at each time point. At least 200 cells were assessed for each sample. Two-tailed unpaired Student's t test was used for statistical comparisons. Data shown are means ±SD (n=5 biologically independent samples per group). p=0.0208 (7h), p=0.0012 (8h) *vs.* untreated (no drug).

(g) *C. albicans*-induced BMDM death assessed by LDH cytotoxicity assay. NSA treated and untreated BMDMs were incubated with *C. albicans* at MOI 10. Relative LDH release was expressed as the percentage LDH activity in supernatants of cultured cells (medium) compared with total LDH (from the medium and cells) and used as an index of cytotoxicity. Two-tailed unpaired Student's t test was used for statistical comparisons. Data shown are means ±SD (n=3 for 0h, 4 for 4h, 8 for 8h, and 4 for12h). p=0.0017 (12h) *vs.* untreated (no drug).

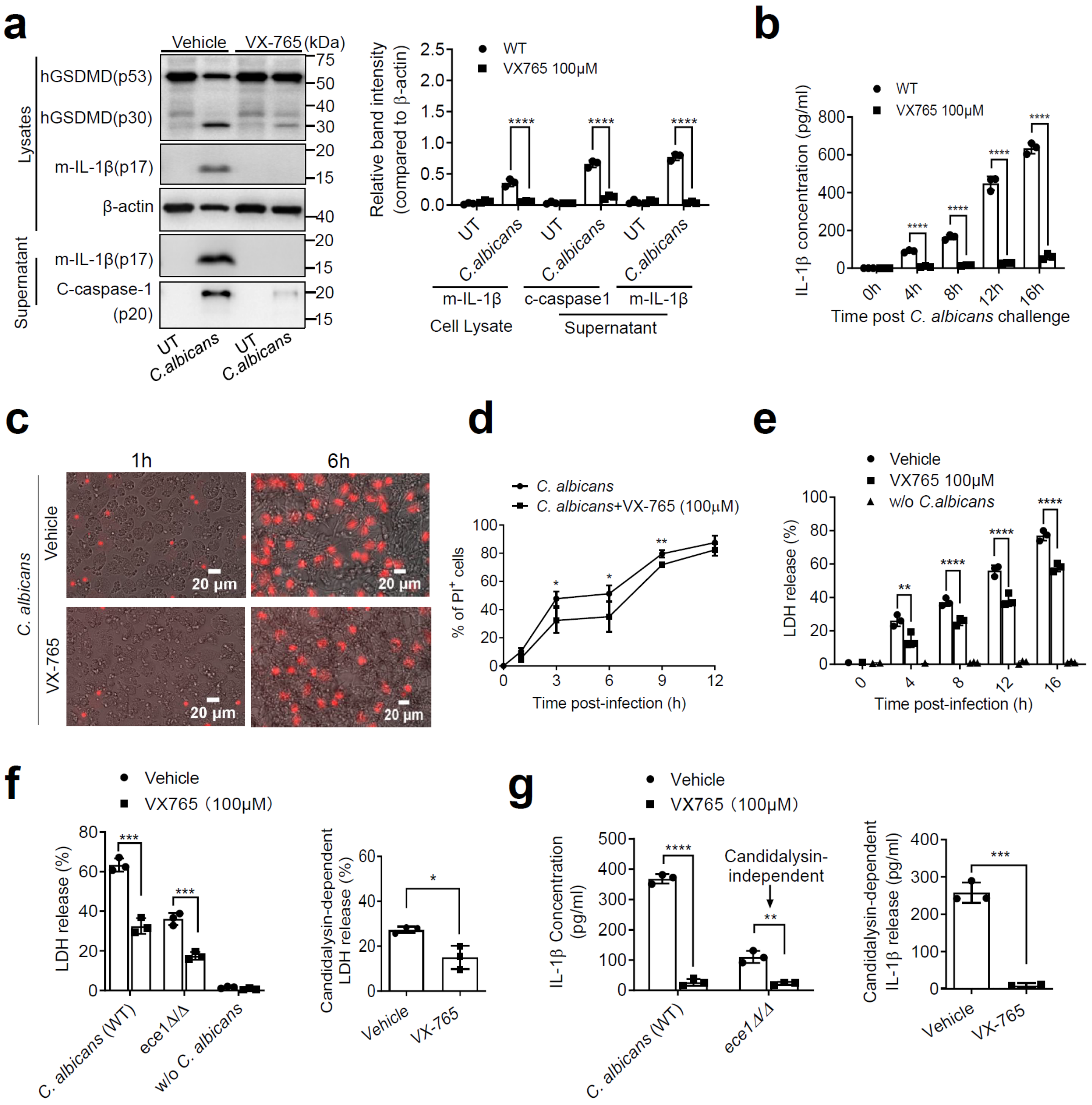
a
Vehicle
VX-765
(kDa)
Lysates
Supernatant
hGSDMD(p53)
hGSDMD(p30)
m-IL-1β(p17)
β-actin
m-IL-1β(p17)
C-caspase-1 (p20)
UT
C.albicans
UT
C.albicans
Relative band intensity (compared to β-actin)
WT
VX765 100μM
m-IL-1β
c-caspase1
m-IL-1β
Cell Lysate
Supernatant
b
IL-1β concentration (pg/ml)
Time post C. albicans challenge
c
1h
6h
C. albicans
Vehicle
VX-765
20 μm
d
C. albicans
C. albicans+VX-765 (100μM)
% of PI+ cells
Time post-infection (h)
e
Vehicle
VX765 100μM
w/o C.albicans
LDH release (%)
Time post-infection (h)
f
Vehicle
VX765（100μM）
LDH release (%)
C. albicans (WT)
ece1Δ/Δ
w/o C. albicans
Candidalysin-dependent LDH release (%)
Vehicle
VX-765
g
Vehicle
VX765（100μM）
IL-1β Concentration (pg/ml)
Candidalysin-independent
C. albicans (WT)
ece1Δ/Δ
Candidalysin-dependent IL-1β release (pg/ml)
Vehicle
VX-765

Supplementary Figure 9. Treatment with caspase-1 inhibitor VX-765 alleviates *C. albicans*-induced death of human macrophages

(a) GSDMD cleavage was blocked by VX-765. VX-765 treated and untreated human monocyte derived macrophages (hMDMs) were infected with WT *C. albicans* for 6 h at MOI 50. Full-length (p53) hGSDMD, cleaved (p30) hGSDMD, mature IL-1β (m-IL-1β), and actin in the cell lysates, as well as m-IL-1β and cleaved caspase-1 (C- caspase-1) in the supernatant, were detected by western blotting. The figure shows the result of a representative experiment that was repeated three times. The intensities of protein bands were quantified using ImageJ. UT, uninfected. Two-tailed unpaired Student's t test was used for statistical comparisons. Data shown are means ±SD (n=3 biologically independent samples). p<0.0001 *vs.* untreated (Vehicle). Source data are provided as a Source Data file.

(b) IL-1β secretion by hMDMs. VX-765 treated and untreated hMDMs were infected with *C. albicans* (MOI=10) for the indicated time periods. IL-1β in culture supernatants was measured by ELISA. Two-tailed unpaired Student's t test was used for statistical comparisons. Data shown are means ±SD (n=3 biologically independent samples per group). p<0.0001 *vs.* untreated (Vehicle).

(c) VX-765 treated and untreated hMDMs were incubated with *C. albicans* at MOI 10. Dying cells were stained with propidium iodide (PI) (red) (200 ng/mL). Images were acquired at indicated time points using 20× dry lens. Shown are representative images at the indicated time points. Scale bars represent 20 μm. Experiments were repeated three times.

(d) The percentage of PI-positive hMDMs calculated at each time point. At least 200 cells were assessed for each sample. Two-tailed unpaired Student's t test was used for statistical comparisons. Data shown are means ±SD (n=4 biologically independent samples per group). p=0.0239 (3h), p=0.0381 (6h), p=0.0023 (9h) *vs.* untreated (no drug).

(e) *C. albicans*-induced hMDM death assessed by LDH cytotoxicity assay. VX-765 treated and untreated hMDMs were incubated with *C. albicans* at MOI 10. Relative LDH release was expressed as the percentage LDH activity in supernatants of cultured cells (medium) compared with total LDH (from the medium and cells) and used as an index of cytotoxicity. Two-tailed unpaired Student's t test was used for statistical comparisons. Data shown are means ±SD (n=3 per group). Results are representative of data from three biological replicates. p=0.0239 (4h), p<0.0001 (8h, 12h, 16h) *vs.* untreated (no drug).

(f) Treatment with VX-765 alleviated both Candidalysin-dependent and -independent *C. albicans*-induced death of human macrophages. VX-765 treated and untreated hMDMs were incubated with WT or candidalysin-deficient (*ece1Δ/Δ*) *C. albicans* at MOI 10 for 12 h. Relative LDH release was assessed as described in Fig.3f. Candidalysin-dependent LDH release was calculated by subtracting the level of LDH release induced by candidalysin-deficient (*ece1Δ/Δ*) mutants from that induced by WT *C. albicans*. Two-tailed unpaired Student's t test was used for statistical comparisons. Data shown are means ±SD (n=3 biologically independent samples per group). p=0.0005 (WT *C. albicans*), p=0.0009 (*ece1Δ/Δ*) *vs.* untreated (Vehicle).

(g) VX-765 treatment suppressed both candidalysin-dependent and -independent IL-1β secretion. VX-765 treated and untreated hMDMs were infected with WT or candidalysin-deficient (*ece1Δ/Δ*) *C. albicans* (MOI=10) for 12 h. IL-1β in culture supernatants was measured by ELISA. Candidalysin-dependent IL-1β release was calculated by subtracting the level of IL-1β release induced by candidalysin-deficient (*ece1Δ*/Δ) mutants from that induced by WT *C. albicans*. Two-tailed unpaired Student's t test was used for statistical comparisons. Data shown are means ±SD (n=3 biologically independent samples per group). For IL-1β concentration, p<0.0001 (WT *C. albicans*), p=0.0017 (*ece1Δ/Δ*) *vs.* untreated (Vehicle). For candidalysin-independent IL-1β release, p=0.0001 *vs.* untreated (Vehicle).

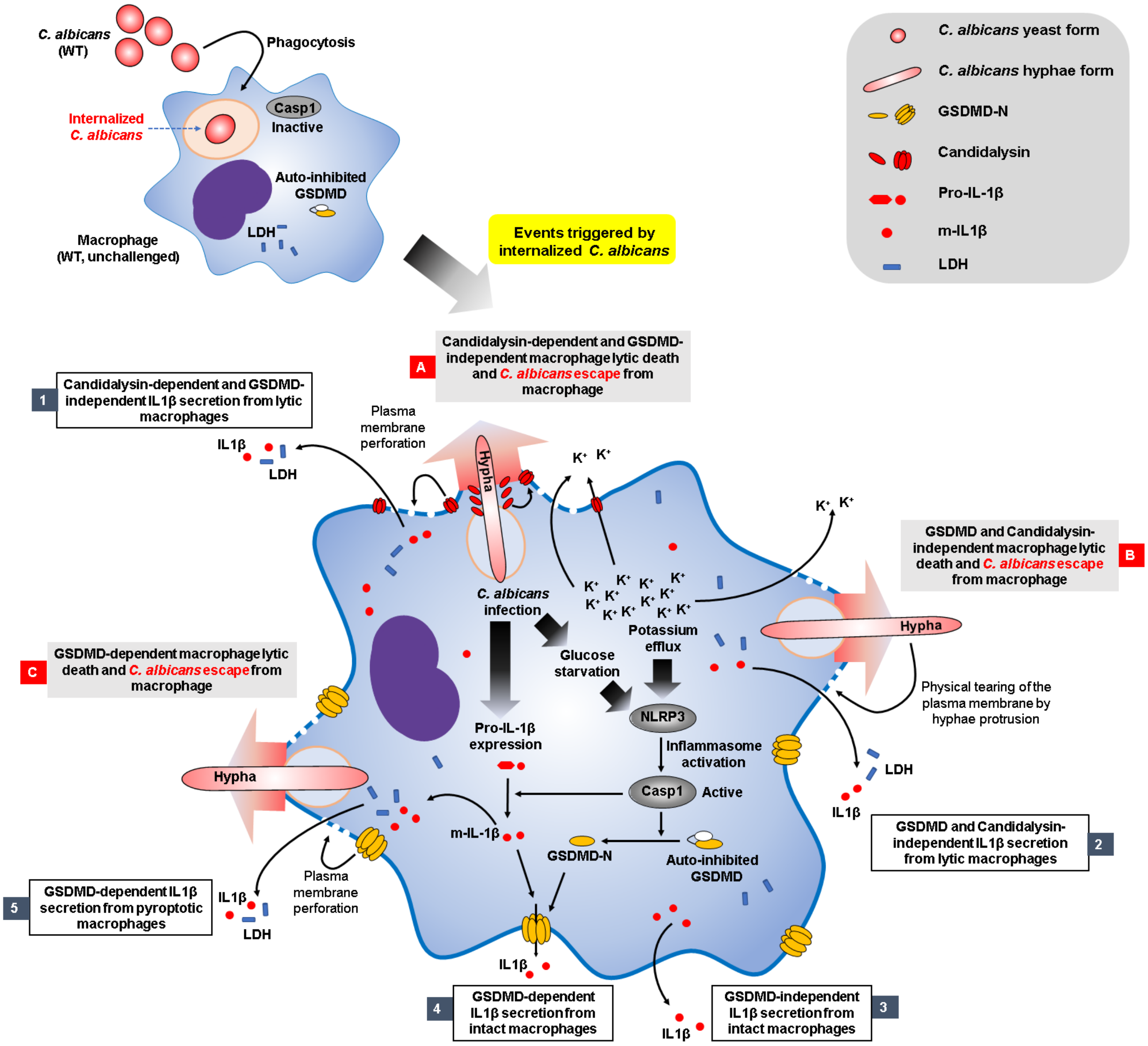

C. albicans	Macrophage	IL1β production		C. albicans escape	
		Effects	Mechanisms	Effects	Mechanisms
WT	WT	Maximum *C. albicans*–induced IL1β secretion	1 2 3 4 5	Maximum *C. albicans* escape from macrophages	A B C
WT	Casp-1/11 DKO	No IL1β production	Pro-IL1β can not be processed to generate mature IL1β	Inflammasome and GSDMD-dependent escape is suppressed	A B
WT	GSDMD KO	GSDMD-independent IL1β secretion	1 2 3	Inflammasome and GSDMD-dependent escape is suppressed	A B
cph1Δ/Δ efg1Δ/Δ (yeast locked)	WT	Pro-IL1β expression is induced but can not be processed efficiently	Hyphae formation is essential for efficient production of mature IL1β	The engulfed *C. albicans* can not escape	Hyphae formation is essential for *C. albicans* escape
ece1Δ/Δ (Candidalysin Deficient)	WT	Candidalysin-dependent inflammasome activation and IL1β secretion is suppressed	2 3 4 5	Candidalysin-independent inflammasome activation can still activate GSDMD	B C
ece1Δ/Δ (Candidalysin Deficient)	GSDMD KO	IL1β secretion can still be mediated by GSDMD and Candidalysin-independent mechanisms	2 3	*C. albicans* escape now can only be mediated by physical tearing of the plasma membrane by hyphae protrusion	B

Supplementary Figure 10. *C. albicans* escape from macrophages is mediated by multiple mechanisms.

Supplementary References

1. Fonzi WA, Irwin MY. Isogenic strain construction and gene mapping in Candida albicans. *Genetics* 1993, **134**(3)**:** 717-728.

2. Lo HJ, Köhler JR, DiDomenico B, Loebenberg D, Cacciapuoti A, Fink GR. Nonfilamentous C. albicans mutants are avirulent. *Cell* 1997, **90**(5)**:** 939-949.

3. Liu N-N, Flanagan PR, Zeng J, Jani NM, Cardenas ME, Moran GP*, et al.* Phosphate is the third nutrient monitored by TOR in Candida albicans and provides a target for fungal-specific indirect TOR inhibition. *Proceedings of the National Academy of Sciences* 2017, **114**(24)**:** 6346-6351.

4. Gerami-Nejad M, Berman J, Gale CA. Cassettes for PCR-mediated construction of green, yellow, and cyan fluorescent protein fusions in Candida albicans. *Yeast* 2001, **18**(9)**:** 859-864.

5. Uhl MA, Johnson AD. Development of Streptococcus thermophilus lacZ as a reporter gene for Candida albicans. *Microbiology (Reading, England)* 2001, **147**(5)**:** 1189-1195.
