# Supplementary material for: Inflammasome-mediated GSDMD activation facilitates escape of Candida albicans from macrophages
Source: Nat Commun. 2021 Nov 18;12:6699. doi: 10.1038/s41467-021-27034-9 (PMC8602704; doi:10.1038/s41467-021-27034-9)
Supplement: Supplementary file 3 — Description of Additional Supplementary Files [file 41467_2021_27034_MOESM3_ESM.pdf]

Description of Additional Supplementary Files

Supplementary Movie 1 (related to Fig.3c). Time-lapse imaging of WT *C. albicans* escaping from WT or *Gsdmd*$^{-/-}$ macrophages. Experiment was conducted as described in **Fig.3c**. WT and *Gsdmd*$^{-/-}$ BMDMs were incubated with *C. albicans* at MOI 10. Dying cells were stained with propidium iodide (PI) (red) (200 ng/mL). Images were acquired every 5 min for 12 h using a 20x dry lens.

Supplementary Movie 2 (related to Fig.4e). Time-lapse imaging of hyphae-deficient *C. albicans* escaping from macrophages. Experiment was conducted as described in **Fig.4e**. Images were acquired every 5 min for 13 h using a 20x dry lens.

Supplementary Movie 3 (related to Fig.6a). Left panel, Time-lapse imaging of WT or candidalysin-deficient *C. albicans* escaping from WT macrophages. Right panel, Time-lapse imaging of WT or candidalysin-deficient *C. albicans* escaping from *Gsdmd*$^{-/-}$ macrophages. Experiment was conducted as described in **Fig.6a**. Images were acquired every 5 min for 12 h using a 20x dry lens.

Supplementary Movie 4 (related to Fig.8b). Time-lapse imaging of WT *C. albicans* escaping from WT or *Casp-1/11*$^{-/}$ macrophages. Experiment was conducted as described in **Fig.8b**. Images were acquired every 5 min for 6 h using a 20x dry lens.

Supplementary Movie 5 (related to Fig.8g). Time-lapse imaging of *C. albicans* escaping from macrophages in the presence of KCl. Experiment was conducted as described in **Fig.8g**. Images were acquired every 5 min for 6 h using a 20x dry lens.
