# Supplementary material for: Inflammasome-mediated GSDMD activation facilitates escape of Candida albicans from macrophages
Source: Nat Commun. 2021 Nov 18;12:6699. doi: 10.1038/s41467-021-27034-9 (PMC8602704; doi:10.1038/s41467-021-27034-9)
Supplement: Supplementary file 9 — Reporting summary [file 41467_2021_27034_MOESM9_ESM.pdf]

nature portfolio

Corresponding author(s): Hongbo R. Luo

Last updated by author(s): Sep 20, 2021

Reporting Summary

Nature Portfolio wishes to improve the reproducibility of the work that we publish. This form provides structure for consistency and transparency in reporting. For further information on Nature Portfolio policies, see our Editorial Policies and the Editorial Policy Checklist.

Statistics

For all statistical analyses, confirm that the following items are present in the figure legend, table legend, main text, or Methods section.

n/a	Confirmed	
☐	☒	The exact sample size (*n*) for each experimental group/condition, given as a discrete number and unit of measurement
☐	☒	A statement on whether measurements were taken from distinct samples or whether the same sample was measured repeatedly
☐	☒	The statistical test(s) used AND whether they are one- or two-sided<br>*Only common tests should be described solely by name; describe more complex techniques in the Methods section.*
☐	☒	A description of all covariates tested
☐	☒	A description of any assumptions or corrections, such as tests of normality and adjustment for multiple comparisons
☐	☒	A full description of the statistical parameters including central tendency (e.g. means) or other basic estimates (e.g. regression coefficient) AND variation (e.g. standard deviation) or associated estimates of uncertainty (e.g. confidence intervals)
☐	☒	For null hypothesis testing, the test statistic (e.g. *F*, *t*, *r*) with confidence intervals, effect sizes, degrees of freedom and *P* value noted<br>*Give P values as exact values whenever suitable.*
☒	☐	For Bayesian analysis, information on the choice of priors and Markov chain Monte Carlo settings
☒	☐	For hierarchical and complex designs, identification of the appropriate level for tests and full reporting of outcomes
☒	☐	Estimates of effect sizes (e.g. Cohen's *d*, Pearson's *r*), indicating how they were calculated

Our web collection on statistics for biologists contains articles on many of the points above.

Software and code

Policy information about availability of computer code

Data collection	For mouse samples, bone marrow-derived macrophages were prepared following the protocol described in the paper of Mondal S et al. (reference 5). For human samples, Human peripheral blood mononuclear cells (hPBMC) were isolated using a Ficoll density gradient medium (LymphoprepTM, Stem cell technologies: Catalog #07801) following a protocol provided by the manufacturer. Subsequently, human monocytes were isolated from hPBMCs using EasySep™ Human Monocyte Isolation Kit (Stem cell technologies: Catalog #19359) according to the manufacturer's protocol. To differentiate monocytes into hMDMs, the isolated monocytes were resuspended in IMDM GlutaMAX Supplement culture medium (ThermoFisher: 31980-030) supplemented with 10% FBS, and 40 ng/ml recombinant human M-CSF. Cells were seeded at 1.5×10^6 in a 6-well plate with 2 ml culture medium per well and cultured for 6 days. The medium was changed on day 3, and floating non-adhesive cells were washed away during the medium change. hMDMs were collected from the plate 6 days after human M-CSF treatment. For western blotting samples, at each time point, BMDMs were lysed immediately with cell lysis buffer (2× Laemmli sample Buffer, Bio-Rad, Hercules, CA) containing 2-mercaptoethanol (Bio-Rad) and protease inhibitor cocktail (Cell Signaling). Proteins in the supernatants were precipitated with chloroform/methanol (1:4) and then dissolved in the same lysis buffer. Samples were denatured by boiling at 100ºC for 5 min and transferred to ice. After brief sonication (5-10 s), 15 µl of sample was subjected to 4-15% gradient SDS-PAGE.
Data analysis	Flow cytometry data were analyzed using Flowjo V10.5.<br>Survival data and other statistical data were analyzed using Prism (GraphPad Software, La Jolla, CA).<br>DsRed filter channel images were processed using NIS-Elements software and cellSens Dimension V2.<br>Confocal microscopy data and western blotting data were analyzed using Image J.<br>Immunoreactivity was detected using an ImageQuant LAS-4000 (GE, Fairfield, CT).

For manuscripts utilizing custom algorithms or software that are central to the research but not yet described in published literature, software must be made available to editors and reviewers. We strongly encourage code deposition in a community repository (e.g. GitHub). See the Nature Portfolio guidelines for submitting code & software for further information.

Data

Policy information about availability of data

All manuscripts must include a data availability statement. This statement should provide the following information, where applicable:

- Accession codes, unique identifiers, or web links for publicly available datasets
- A description of any restrictions on data availability
- For clinical datasets or third party data, please ensure that the statement adheres to our policy

All data needed to evaluate the conclusions in the paper, including data associated with main figures and supplementary figures, are available within the article or in the online-only Data Supplement. This study did not generate any other digitally accessible data or code. Source data will be provided with this paper. Plasmids and C. albicans strains generated in this study are available from the Lead Contact with a completed Materials Transfer Agreement. This study did not generate any other unique distributable reagents.

Field-specific reporting

Please select the one below that is the best fit for your research. If you are not sure, read the appropriate sections before making your selection.

☒ Life sciences ☐ Behavioural & social sciences ☐ Ecological, evolutionary & environmental sciences

For a reference copy of the document with all sections, see nature.com/documents/nr-reporting-summary-flat.pdf

Life sciences study design

All studies must disclose on these points even when the disclosure is negative.

Sample size	Sample size was determined based on the knowledge on good sample size to ensure adequate data for reliable assessments. Sample sizes are always indicated in figure legends or methods section. For in vitro experiments were repeated at least three times. For in vivo experiments, for reliable statistical analysis, at least five mice from each genotype or treatment group were utilized for each data point.
Data exclusions	No data was excluded from the analysis.
Replication	All experiments were performed at least three times independently and successfully reproduced.
Randomization	No randomization was used in this study, because all experimental mice(WT) were purchased from the Jackson laboratory and kept under the same environment. Corresponding sex- and age-matched WT mice were used as controls in all experiments performed with KO mice.
Blinding	The investigators were blinded to group allocation during data collection and analysis.

Reporting for specific materials, systems and methods

We require information from authors about some types of materials, experimental systems and methods used in many studies. Here, indicate whether each material, system or method listed is relevant to your study. If you are not sure if a list item applies to your research, read the appropriate section before selecting a response.

Materials & experimental systems

n/a	Involved in the study	
☐	☒	Antibodies
☒	☐	Eukaryotic cell lines
☒	☐	Palaeontology and archaeology
☐	☒	Animals and other organisms
☐	☒	Human research participants
☒	☐	Clinical data
☒	☐	Dual use research of concern

Methods

n/a	Involved in the study	
☒	☐	ChIP-seq
☐	☒	Flow cytometry
☒	☐	MRI-based neuroimaging

Antibodies

Antibodies used	For flow cytometry analysis, PE-Cy7-conjugated CD45 (BioLegend, cat 103114, clone 30 F11), PE-conjugated F4/80 (BioLegend, cat 123109, clone BM8), and APC-conjugated CD11b (BioLegend, cat 101212, clone M1/70) antibodies were used. For western blotting, mouse anti-GSDMD antibody (ab209845, 1:500; Abcam, Cambridge, UK), mouse anti-IL-1-β/IL-1F2 antibody (AB-401-NA, 1:1000; R&D Systems, Minneapolis, MI), anti-total IL-1β antibody (D3H1Z) (#12507, 1:1000; Cell Signaling Technology, Danvers, MA), anti-cleaved-IL-1β (Asp117) antibody (52718S, 1:1000; Cell Signaling Technology), anti-human Gasdermin D (E9S1X) antibody (39754S 1:1000; Cell Signaling Technology), anti-human Cleaved Gasdermin D (Asp275) (E7H9G) (36425S 1:1000; Cell Signaling Technology), anti-human Cleaved-IL-1β (Asp116) (D3A3Z) (83186S 1:1000; Cell Signaling Technology), anti-human Cleaved Caspase-1 (Asp297)

	(D57A2) (4199S 1:1000; Cell Signaling Technology), or anti-β-actin antibody (A2228, 1:1000; Sigma Aldrich, St. Louis, MO) were used. For cell death, Propidium Iodide (Thermo Fisher Scientific, Cat P3566) was used.
Validation	All antibodies used in this study are commercially available from well-known companies. All antibodies used for flow cytometry are well-established lineage marker antibodies. Staining patterns were consistent with the manufacturer product information as well as published data. All antibodies used for western blotting are well-established antibodies. Staining pattern and size of molecular weight were consistent with the manufacturer product information as well as published data.<br>For flow cytometry analysis,<br>PE-Cy7-conjugated CD45 (BioLegend, cat 103114, clone 30 F11)-manufacturer's validation<br>PE-conjugated F4/80 (BioLegend, cat 123109, clone BM8)-manufacturer's validation<br>APC-conjugated CD11b (BioLegend, cat 101212, clone M1/70)-manufacturer's validation<br>For western blotting,<br>mouse anti-GSDMD antibody (ab209845, 1:500; Abcam, Cambridge, UK)-manufacturer's validation<br>mouse anti-IL-1-β/IL-1F2 antibody (AB-401-NA, 1:1000; R&D Systems, Minneapolis, MI)-manufacturer's validation<br>anti-total IL-1β antibody (D3H1Z) (#12507, 1:1000; Cell Signaling Technology, Danvers, MA)-manufacturer's validation<br>anti-cleaved-IL-1β (Asp117) antibody (52718S, 1:1000; Cell Signaling Technology)-manufacturer's validation<br>anti-human Gasdermin D (E9S1X) antibody (39754S 1:1000; Cell Signaling Technology)-manufacturer's validation<br>anti-human Cleaved Gasdermin D (Asp275) (E7H9G) (36425S 1:1000; Cell Signaling Technology)-manufacturer's validation<br>anti-human Cleaved-IL-1β (Asp116) (D3A3Z) (83186S 1:1000; Cell Signaling Technology)-manufacturer's validation<br>anti-human Cleaved Caspase-1 (Asp297) (D57A2) (4199S 1:1000; Cell Signaling Technology)-manufacturer's validation<br>anti-β-actin antibody (A2228, 1:1000; Sigma Aldrich, St. Louis, MO)-manufacturer's validation<br>For cell death,<br>Propidium Iodide (Thermo Fisher Scientific, Cat P3566)-manufacturer's validation

Animals and other organisms

Policy information about studies involving animals; ARRIVE guidelines recommended for reporting animal research

Laboratory animals	Gsdmd-/- mice (in a C57BL/6J background) were generated as previously described (reference 1). Casp1/11-/- (in a C57BL/6NJ background), C57BL/6J WT, and C57BL/6NJ WT mice were purchased from The Jackson Laboratory (Bar Harbor, ME). Eight-to-twelve week-old male or female mice were used in all experiments. All mice were fed and watered ad libitum with consistent access to food and water, and all mice were housed in a facility kept at ambient temperature and humidity with 12 hr light/12 hr dark cycles.
Wild animals	No wild animal was used in this study.
Field-collected samples	No field collected samples were used.
Ethics oversight	All animal experiments were conducted in accordance with the Animal Welfare Guidelines of the Children's Hospital Boston. The Children's Hospital Animal Care and Use Committee approved and monitored all procedures.

Note that full information on the approval of the study protocol must also be provided in the manuscript.

Human research participants

Policy information about studies involving human research participants

Population characteristics	donor: healthy male or females about 25-35 years old.
Recruitment	Healthy people undergoing routine medical check-up at participating site were asked whether they were interested in participating in this study. No incentives were provided and there is no bias in selection.
Ethics oversight	The Ethics Committee of Children's Hospital Boston approved the study protocol. All participating blood donors provided written informed consent for sample collection and data analysis.

Note that full information on the approval of the study protocol must also be provided in the manuscript.

Flow Cytometry

Plots

Confirm that:

- [x] The axis labels state the marker and fluorochrome used (e.g. CD4-FITC).
- [x] The axis scales are clearly visible. Include numbers along axes only for bottom left plot of group (a 'group' is an analysis of identical markers).
- [x] All plots are contour plots with outliers or pseudocolor plots.
- [x] A numerical value for number of cells or percentage (with statistics) is provided.

Methodology

Sample preparation	Mice (WT and Gsdmd-/-) were intravenously challenged with 3 × 105 CFU WT (GFP+) or candidalysin-deficient (ece1Δ/Δ) C. albicans. Three days after C. albicans infection, kidneys were homogenized using 40 µm cell strainers. Red blood cells were lysed with 1 ml ACK lysis buffer (Gibco) for 5 min at room temperature. Cells were then stained with PE-Cy7-conjugated CD45 (BioLegend), PE-conjugated F4/80 (BioLegend), and APC-conjugated CD11b (BioLegend) antibodies for 30 min. For cells isolated from WT (GFP+) C. albicans-infected mice, macrophages containing trapped C. albicans were recognized by GFP fluorescence. For cells isolated from candidalysin-deficient (ece1Δ/Δ) C. albicans-infected mice, the samples were fixed and permeabilized (BD Biosciences) after surface marker staining, and then stained with FITC-conjugated anti-C. albicans antibody (thermofisher) for 1h.
Instrument	Flow cytometry was performed on the LSRFortessa (BD Biosciences) instrument.
Software	Flow cytometry data were analyzed with FlowJo software. https://www.flowjo.com/solutions/flowjo/downloads/
Cell population abundance	We were interested in the macrophages containing trapped C. albicans that were GFP+ cells in macrophages(CD45+F4/80 +CD11b+).
Gating strategy	Gating strategies for specific cell populations were illustrated in Figure S5b. Primary gates are the CD45+ cells. In the CD45+ cell gates, we next gated F4/80+CD11b+ cells (macrophages). Finally, we were interested in the macrophages containing trapped C. albicans that were GFP+ cells in macrophages.

☒ Tick this box to confirm that a figure exemplifying the gating strategy is provided in the Supplementary Information.
